# Defectors’ intolerance of others promotes cooperation in the repeated public goods game with opting out

**DOI:** 10.1038/s41598-020-76506-3

**Published:** 2020-11-11

**Authors:** Vlastimil Křivan, Ross Cressman

**Affiliations:** 1 Department of Mathematics, Faculty of Science, University of South Bohemia, 370 05 Ceske Budejovice, Czech Republic; 2 Department of Mathematics, Wilfrid Laurier University, Waterloo, ON Canada; 3 Czech Academy of Sciences, Biology Centre, 370 05 Ceske Budejovice, Czech Republic

**Keywords:** Evolutionary theory, Human behaviour, Applied mathematics, Social evolution

## Abstract

The theoretical and experimental research on opting out (also called conditional dissociation) in social dilemmas has concentrated on the effect this behavior has on the level of cooperation when used against defectors. The intuition behind this emphasis is based on the common property of social dilemmas that individuals are worse off the more their opponents defect. However, this article shows clearly that other opting out mechanisms are better at increasing cooperative behavior. In fact, by analyzing the stable Nash equilibria for the repeated multi-player public goods game with opting out, our results provide a strong argument that the best opting out rule is one whereby the only groups that voluntarily stay together between rounds are those that are homogeneous (i.e., those groups that are either all cooperators or all defectors), when these groups stay together for enough rounds. This outcome emerges when defectors are completely intolerant of individuals who cooperate (e.g., defectors exhibit xenophobic behavior toward cooperators) and so opt out whenever their group has a cooperator in it. The strong preference by defectors to be with like-minded individuals causes all heterogeneous groups to disband after one round.

## Introduction

In the classic single-round public goods game (PGG) where groups consist of *m* players, contributing nothing to the public good (i.e., defecting) is the only Nash equilibrium (NE)^1,2^. Thus PGG provides a well-known example of a social dilemma in that groups are best off (i.e., receive the highest payoff) if all members contribute their entire endowment *E* (i.e., cooperate) rather than defect. By backward induction, the same result holds for the repeated version of PGG when the number of rounds played by all groups is the same^3,4^. One way to resolve this dilemma is to assume that, in a given round, each group disbands with a certain probability $0<\rho <1$, in which case it is only the expected number of rounds $1/\rho$ played by all groups that is the same. Then other NE outcomes emerge including all individuals cooperating or all individuals contributing some of their endowment to the public good, especially if there is a mechanism for players to punish defectors or to not participate in the game^5–7^.

Here, we explore another way to promote cooperative behavior in the repeated PGG whereby members are allowed to opt out of their group depending on the total amount contributed by group members. In fact, we consider a simplified set of strategies where each individual is either a Defector or Cooperator for the entire game. In particular, an individual’s action (i.e., Defect or Cooperate) in a given round cannot depend on what actions were taken by group members in previous rounds, thereby considerably reducing the number of strategies often used in repeated games^8^. In our simplified game, the decision whether to opt out is then a function of the number of Cooperators in their group. If any group member opts out in a given round, the group disbands. Otherwise, the group disbands with probability $0<\rho <1$. All individuals from disbanded groups form new *m*-player groups at random and join the continuing groups for the next round.

As we will see, opting-out influences expected payoffs individuals receive through its effect on group distributions and consequently the game’s expected outcome. Opting out (also called conditional dissociation,^9^) against Defectors, that leads to a positive assortment of Cooperators, is known to promote cooperation in the two-player repeated Prisoner’s dilemma (PD) game (e.g.,^10–15^) and in the repeated two-player PGG^16^. On the other hand, it is also known that individuals who are cooperative in PD games may not be so in PGG^17^.

The questions of most interest to us are whether opting out continues to promote cooperation in the *m*-player repeated PGG and, if so, what opting out rules accomplish this and why individuals adopt them.

## Results

First, we briefly summarize the simplified version of the single-round PGG. Let *E* be each player’s endowment in the single round where all groups consist of *m* players ($m\ge 2$). The two strategies are to Defect (D) and Cooperate (C) by contributing 0 and *E* respectively to the public good. In a group of *k* Cooperators, the single-round payoffs to a Cooperator and a Defector are

1 $$\begin{aligned} \pi _C(k)&= \frac{k r E}{m}\\ \pi _D(k)&= E+\frac{k r E}{m} \end{aligned}$$

where $r>1$ is the enhancement factor and $k=0,\dots ,m$. (Technically, $\pi _C(k)$ is undefined for $k=0$ and $\pi _D(k)$ is undefined for $k=m$). If $r<m$, then it is always better for a focal individual to play D than C when the strategies of the rest of the group members are fixed (i.e., in a group consisting of *k* Cooperators it pays off for a Cooperator to switch to defection because its payoff in the group with the remaining $k-1$ Cooperators will then be higher as $\pi _D(k-1)>\pi _C(k)$ for all $1\le k\le m$). Then all D is the only NE (i.e., all group members contribute nothing to the public good). In the classic (single-round) PGG, it is typically assumed that $1<r<m$ as this leads to a conflict between maximizing total group payoff and NE behavior (i.e., optimizing individual payoff). Indeed, since $r>1$, payoffs to the all C group is higher than to the all D group (i.e., $\pi _C(m)>\pi _D(0)$) and, since $r<m$, the only NE for the game is all D. In fact, all D is a strict NE since every member of the all D group receives higher payoff playing D than switching to C. It is well-known^18^ that all D is then a stable outcome under standard game dynamics such as the replicator equation. On the other hand, if $r>m$, then it is always better to play C than D (i.e., $\pi _D(k-1)<\pi _C(k)$) and so the NE is all C. Finally, if $r=m$, then there is no single NE since every group composition of Cooperators and Defectors is a NE.

In our repeated PGG game, where each individual is either a Cooperator or a Defector for the entire game, each individual has an opting out rule that depends on the number of Cooperators in this player’s group. We further simplify our repeated PGG game by assuming that all Cooperators have the same opting out rule for the entire game as do all Defectors. Our *m*-player PGG with opting out then has two (pure) strategies, C along with its opting out rule and D along with its opting out rule. In this repeated game, either at least one group member opts out after the first round (in which case, the group disbands) or the group plays for an expected number $\tau =1/\rho$ of rounds. That is, for each number of Cooperators in the group, $k=0,\dots ,m$, the expected number of rounds $\tau _k$ played by this group is either 1 or $\tau$. Thus, there are $2^{m+1}$ possible *m*-player PGG with opting out corresponding to all combinations of $\{\tau _0,\dots ,\tau _m\}$ where $\tau _k$ is either 1 or $\tau$ for $k=0,\dots ,m$. The classic repeated PGG that corresponds to $\{\tau _0,\dots ,\tau _m\}=\{\tau ,\dots ,\tau \}$ is also considered to be a PGG with opting out by allowing the case where individuals never opt out. There may be more than one choice of opting out rules for C and D that generate the same PGG with opting out. For example, with $m=2$, if only Cooperators opt out and this occurs if and only if their group has 1 Cooperator, then $\tau _0=\tau _2=\tau , \tau _1=1$. The same game is generated if only Defectors opt out and this occurs if and only if their group has 1 Cooperator.

To complete the description of our *m*-player PGG with opting out, the payoff structure of the game must be specified. In this repeated PGG, players receive payoffs given by (1) in each round that their group has *k* Cooperators. For fixed population size $N>0$, where *N* is taken as a large multiple of *m*, individual fitness for a Cooperator (respectively, Defector) is their expected payoff per round $\Pi _C$ (respectively, $\Pi _D$) evaluated at the equilibrium group distribution. Here we define fitness as the individual’s payoff in our repeated game. We avoid labelling this as payoff so as not to confuse this term with payoff in the single-round game. This fitness, that depends on both *m* and $\tau$ through the group distributions given by the opting out rule, is then a function of the total number $N_C$ and $N_D$ of Cooperators and Defectors, respectively, where $N=N_C+N_D$ (see “Methods” section). In fact, our results do not depend on the value of *N* (see subsection Fitness functions in “Methods” section). The two strategies (C, D) together with these two fitness ($\Pi _C$, $\Pi _D$) functions define an *m*-player symmetric two-strategy evolutionary (population) game. There is at least one NE in each such game (i.e., one of the two pure strategies or a mixture of them that is a best reply against itself). We are particularly interested in those NE that are stable (in the sense that nearby trajectories of standard evolutionary dynamics such as the replicator equation converge to this NE) since unstable NE are not expected to correspond to the game’s outcome (see subsection Numerical methods when $m>2$ in “Methods” section).

This same approach has been used to analyze the two-player Prisoner’s dilemma (PD) game^12,15^ with opting out. Here, if players opt out whenever their opponent defects, defect remains a (strict) NE. However, stable coexistence of cooperators and defectors also emerges when $\rho$ is sufficiently small (i.e., when the maximum expected number of rounds played by a group is sufficiently large). In fact, as $\rho$ approaches 0, this coexistence outcome converges to all C and attracts almost all initial population configurations under standard evolutionary dynamics. As we will see, similar results occur for the multi-player PGG.

We note that other theoretical studies have examined the evolutionary stability of opting out rules by incorporating several of them into a single multi-strategy game. These include a three-strategy repeated PGG with opting out where Cooperators have two different opting out rules^19^ and a multi-strategy repeated PD game with opting out where individual choices in later rounds depend on what group members have already played in previous rounds^20^ (see also^9^). A stable NE of our simplified game need no longer be stable (or even a NE) when the strategy set is enlarged in these ways. Such considerations are beyond the scope of this article.

In our repeated PGG games, all D and all C are strict NE if and only if this is also the case for the single-round PGG (see subsection Analytic methods when $m=2$ in “Methods” section). That is, all D (respectively, all C) is a strict NE if and only if $r<m$ (respectively, $r>m$). In particular, in the case of most interest for PGG (i.e., $r<m$), the undesirable result that all D is a stable NE remains as a dilemma in our simplified repeated PGG. Furthermore, we cannot get all C as the NE as is possible in the typical repeated PGG when individual strategies can depend on actions taken by group members in previous rounds or on the group’s size^21^. Since we cannot achieve complete cooperation when $r<m$, of more interest is the existence and stability of NE where there is coexistence of Cooperators and Defectors. Also of interest are the opting out rules that generate coexistence NE and the level of cooperation attainable at them. The following two subsections provide results on this for the *m*-player PGG with opting out when $m=2$ and $m>2$ respectively.

Before doing so, we note that the two extreme opting out rules to always opt out and to never opt out generate the single-round PGG and two-strategy classic repeated PGG respectively. That is, the expected number of rounds each group plays is one and $\tau$ rounds respectively. Since both these games have the same fitness functions (see subsection All groups play the same expected number of rounds in “Methods” section), their only NE is all D (respectively, all C) if $r<m$ (respectively, $r>m$). This result for PGG with never opting out contrasts with those of the classic repeated PGG where cooperative behavior emerges in experiments^22^ as well as in theoretical models^7^. The reason no NE includes Cooperators in our model is that it is based on only two strategies whereas the typical repeated PGG allows individual choice in later rounds of the same group in the repeated game to depend on what strategies were played by group members in earlier rounds (e.g.,^7^).

### Opting out in the two-player public goods game

**Figure 1:**
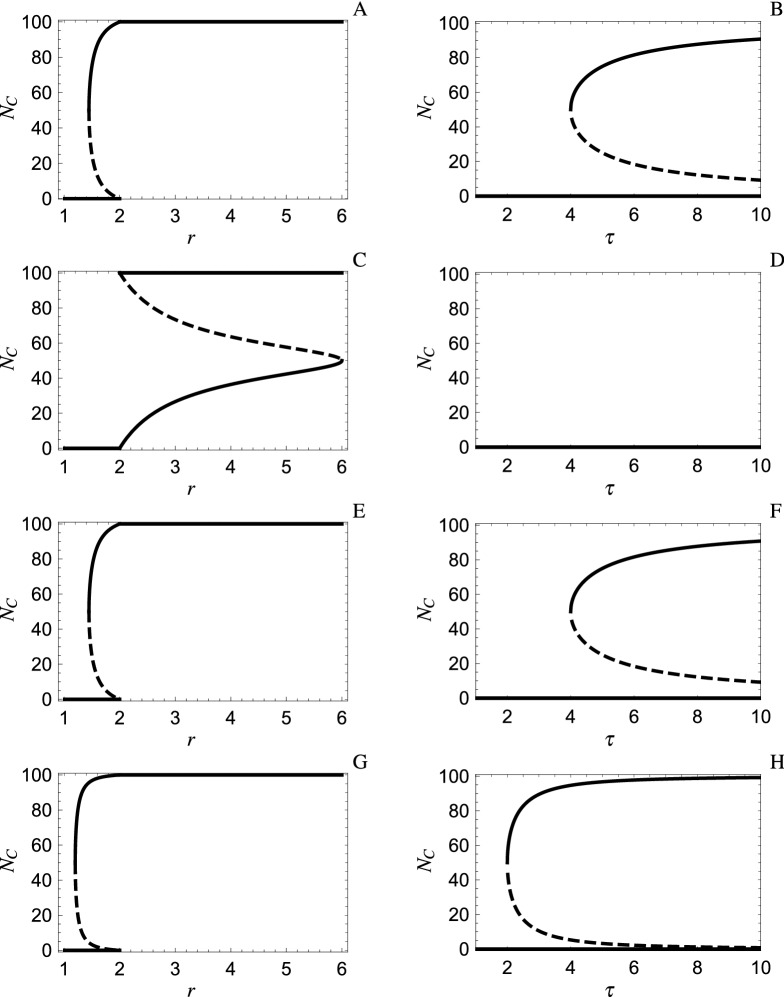
NE of the simplified repeated two-player ($m=2$) PGG with opting out where the expected number of rounds $\tau$ when players do not opt out is 5 (respectively, $\tau >1$) in the left (respectively, right) panels. Solid lines denote stable NE, dashed lines denote unstable NE. The four rows starting at the top correspond to the four rules: opt out if your opponent defects; opt out if your opponent plays the same strategy; opt out if your opponent cooperates; opt out if your opponent plays opposite strategy. Other parameters: $N=100$, $E=1$, $\tau =5$ in left panels and $r=1.5$ in right panels.

The two-player game has been solved analytically by Křivan and Cressman^12^ (see also^23^) for arbitrary single-round payoffs and arbitrary number of rounds $\tau _k$, $k=0,1,2$. Of particular interest for social dilemmas is the emergence of cooperative behavior. Indeed, for the two-player repeated PD where Cooperators give benefit *b* to their opponent at a cost *c* to themselves ($b>c>0$), Křivan and Cressman^12^ (see also^15^) show that, when players opt out if and only if their opponent Defects (i.e., $\tau _0=\tau _1=1, \tau _2=\tau$), stable cooexistence of defection and cooperation emerges when two Cooperators expect to play sufficiently many rounds $\tau$ (specifically, for $\tau >(\frac{b+c}{b-c})^2$). When this same opting out rule is applied to PGG with $r<m=2$, stable coexistence again emerges for $\tau >(\frac{1}{r-1})^2$ (see subsection Analytic methods when $m=2$ of “Methods” section and Fig. 1B).

One motivation of this rule for PD and PGG is based on their single-round payoff matrices. For both games, all players prefer to play against Cooperate. For instance, in the single-round payoff matrix for PGG given by2C has higher payoff paired with C than with D (i.e., $rE>\frac{1}{2}rE$) as does D (i.e., $E+\frac{1}{2}rE>E$). Players then opt out against Defect in the hope of being paired with Cooperate in the next round. Indeed, as confirmed by (8) in Section Analytic methods when $m=2$, this rule does lead to positive assortment among Cooperators when there is a mixture of Cooperators and Defectors in the population (i.e., there are more Cooperator pairs than without opting out for a fixed population distribution) since $\tau _0\tau _2>\tau _1^2$. Furthermore, there is also positive assortment among Defectors. This increase in CC and DD pairs increases the fitness of Cooperators and decreases the fitness of Defectors, resulting in a stable coexistence NE when $r<2$ and $\tau >(\frac{1}{r-1})^2$.

However, this positive assortment is also a consequence of the counterintuitive rule that opts out if and only if the opponent Cooperates (i.e., $\tau _0=\tau , \tau _1= \tau _2=1$). Indeed, these two games are identical (i.e., they have the same fitness functions and so the same stable NE) as illustrated in Fig. 1, where panels A and B are the same as panels E and F, and verified in the “Methods” section. The counterintuitive rule is difficult to justify based on payoffs. It is as if the players are in a parallel universe where individuals prefer to be with a Defector. In fact, results from game experiments^15^ show that participants seldom opt out against Cooperate compared to their propensity to do so against Defect when they know their opponent’s behavior (C or D) and the consequent payoffs in the current round. On the other hand, the fact that this counterintuitive rule also promotes cooperation means that we need to consider other opting out rules as well that may have better justifications based on individual behavior. Some of these rules are actually bad for cooperation. For example, opting out if your opponent exhibits your behavior (i.e., $\tau _0= \tau _2=1, \tau _1=\tau$) generates a PGG with opting out where the only NE for all $\tau \ge 1$ is all D when $r<2$ (Fig. 1C,D). Note that here, and elsewhere, the pronoun “you” refers to a focal individual in the population. For example, “opting out if your opponent exhibits your behavior” is then a more convenient way to say that an individual opts out if his/her opponent exhibits the same behavior as this focal individual.

The emphasis in this article is on PGG with opting out when $r<m$ since this is the typical assumption for PGG. On the other hand, our analysis also provides results for $r>m$. For instance, if $r>m=2$, opting out if your opponent does not exhibit your behavior promotes defection (Fig. 1C). That is, without opting out, the only stable NE is all C whereas, with this opting out, an interior stable NE emerges for $2<r<6$.

From Methods, one rule of particular importance when $r<2$ is to opt out if and only if the opponent does not play your strategy (i.e., $\tau _0= \tau _2=\tau , \tau _1=1$). From Fig. 1G, the coexistence of Cooperate and Defect emerges for smaller *r* under this rule than for opting out against Defect (Fig. 1A) and for smaller $\tau$ (cf. panel H vs. B). In fact, this rule is the best among the $2^3=8$ PGG with opting out at promoting cooperative behavior according to the following criteria (see subsection Analytic methods when $m=2$):

1. For fixed $\tau$, it has the lowest value of *r* at which the stable interior NE begins to appear.
2. For fixed *r*, it has the lowest value of $\tau$ at which the stable interior NE begins to appear.
3. For fixed $\tau$ and *r* for which a stable interior mixed NE exists for some other opting out rule, it has a larger domain of attraction (i.e., the set of initial points whose trajectory converges to this NE is a larger interval).
4. For fixed $\tau$ and *r* for which a stable interior mixed NE exists for some other opting out rule, it has a higher individual fitness and higher level of cooperation.

Moreover, it is the best rule in comparison with any choice of $1\le \tau _k\le \tau$ for $k=0,1,2$. Although these games cannot be generated by opting out rules unless all $\tau _k$ equal 1 or $\tau$, they could be enforced by an exogenous agency whose goal is to promote cooperative behavior.

In summary, in order to have the most cooperation possible in the two-player PGG with opting out, it is best for players to ignore the single-round payoffs when deciding whether to opt out and base this decision instead on the homogeneity of their current pair (i.e., opt out if and only if your group is heterogeneous). This opting out rule can be understood as translating a preference to be paired with like-minded individuals (i.e., individuals who play the same strategy). In this regard, it is well-known^24,25^ that individual behavior may result from other preferences than that based exclusively on payoff. Other opting out behaviors also generate this PGG that is best at promoting cooperation. For instance, this outcome occurs if Cooperators never opt out but Defectors are intolerant of Cooperators to such an extent that they refuse to play another round in a group that contains a Cooperator (e.g., Defectors display xenophobic behavior toward Cooperators). The result is reflected in the article’s title and its extension to multi-player PGG is examined in the following section.

The result also raises the question whether previous studies on opting out promoting cooperation (many of them based on the two-strategy repeated PD game) should have concentrated to such an extent on doing so against Defect (e.g.,^9–15^) rather than on some other attribute of individual behavior. In this regard, it is well-documented that certain types of individuals are intolerant of others. For instance, conservatives (right-wing individuals) are more intolerant of liberals (left-wing individuals) than of conservatives (e.g.,^26,27^) and also tend to be less cooperative than liberals (e.g.,^28,29^). If these tendencies manifest themselves through opting out behavior and interactions among liberals and conservatives are modelled as a public goods game (in particular, liberal and conservative strategies correspond to Cooperate and Defect respectively), we have a situation where the attributes of conservatives that are not based on payoff considerations lead to more cooperation in the population. We return to this point in the final Discussion section.

### Opting out in the multi-player ($m>2$) public goods game

When $m>2$, the number of PGG with opting out grows exponentially in terms of group size, making the mathematical analysis more complex. Moreover, far worse from a theoretical perspective, the game cannot be solved analytically when $m>2$ and coexistence NE have to be determined numerically (see subsection Numerical methods when $m>2$). Here, we restrict our discussion for the most part to four-player PGG (i.e., $m=4$) with opting out since the number of such games (i.e., $2^5=32$) remains manageable. Another reason is that most game experiments on PGG (e.g.,^30–32^) assume a group size of four.

**Figure 2:**
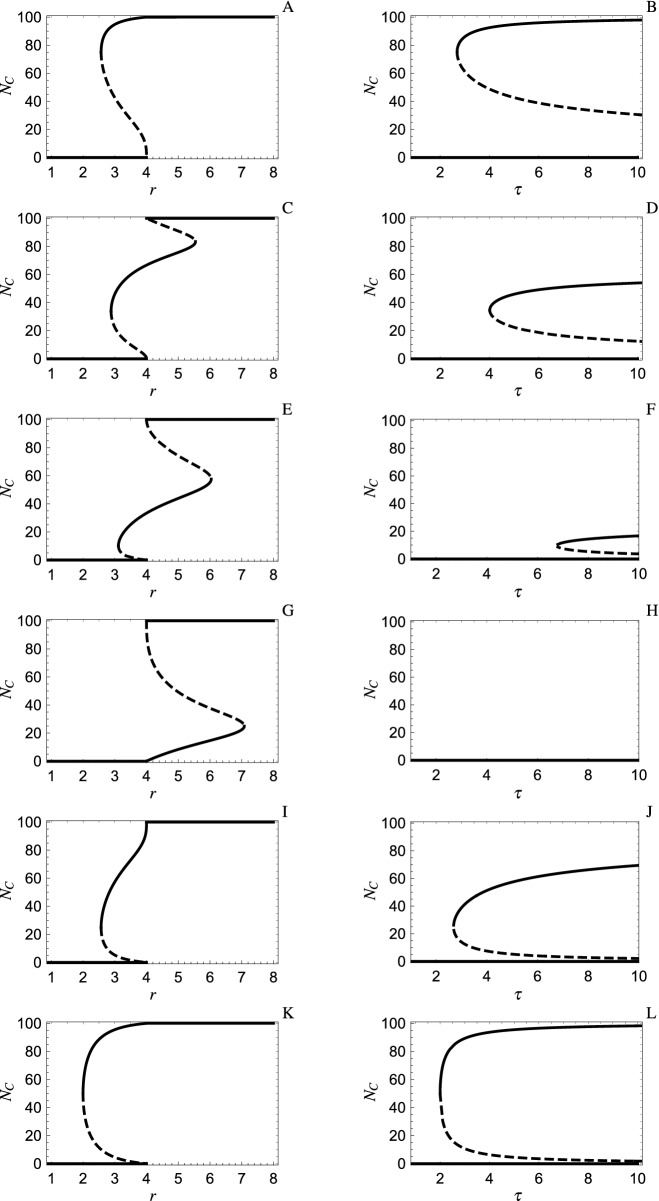
NE of the simplified repeated PGG with opting out for groups of four players ($m=4$) where the expected number of rounds $\tau$ when players do not opt out is 5 (respectively, $\tau >1$) in the left (respectively, right) panels. Solid lines denote stable NE, dashed lines denote unstable NE. For the first four rows starting at the top, the group disbands if and only if it has fewer that $k^*$ Cooperators in it where $k^*=4$ in panels A and B, $k^*=3$ in panels C and D, $k^*=2$ in panels E and F, $k^*=1$ in panels G and H. Row five (panels I and J) assumes that the group disbands if and only if it contains at least one Cooperator. Row six (panels K and L) assumes the group disbands if and only if it is heterogeneous. Other parameters used in simulations: $N=100$, $E=1$, $\tau =5$ in left panels and $r=3$ in right panels.

First, suppose that opting out is based on single-round payoff. It is then intuitively appealing to consider those opting out rules based on a threshold level of Cooperators in your group^19^. Specifically, suppose that if an individual opts out when in a group with *k* Cooperators, s/he will also opt out when in a group with $k-1$ Cooperators since an individual’s single-round payoff increases as the number of Cooperators in the group increases. Under our assumption that all Cooperators use the same opting out rule as do all Defectors, there then exists a threshold $k^*$ such that the group with *k* Cooperators disbands after the first round if and only if $k<k^*$ (i.e., $\tau _k=1$ and $\tau _k=\tau$ if $k<k^*$ and $k\ge k^*$ respectively).

The NE of the resulting games for $m=4$ are provided in Fig. 2 for threshold values $k^*=4$ (panels A, B), $k^*=3$ (panels C, D), $k^*=2$ (panels E, F) and $k^*=1$ (panels G, H). In contrast to the results for two-player games in the previous section, stable coexistence may emerge in the same game for both $r<m$ and $r>m$ (specifically when $k^*=3$ or $k^*=2$ in Fig. 2 panels C and E respectively). Moreover, the two interior NE (when they exist) are no longer symmetric about $N_C=N/2$. More importantly, it is no longer clear which opting out rule among these four based on single-round payoffs is the best at promoting cooperation. For instance, the rule with threshold $k^*=4$ (i.e., the group disbands after the first round unless all members Cooperate) has the lowest value of both *r* (Fig. 2 panel A compared to C, E and G) and $\tau$ (Fig. 2 panel B compared to D, F and H) where a stable coexistence NE exists. This rule also has the highest level of cooperation. On the other hand, threshold $k^*=4$ does not always have the largest domain of attraction for a stable interior NE. The domain of attraction for a stable interior NE in Fig. 2 (when this NE exists for a given $\tau$ and $r<m$) is the vertical interval from the unstable interior NE (i.e., the dashed curve) to $N_C=100$. For instance, as determined numerically by comparing the top four left panels of Fig. 2, the largest domain of attraction (for the parameters used in Fig. 2 for the four-player PGG) is in panel E ($k^*=2$) when $3.11<r<4$, it is in panel C ($k^*=3$) when $2.88<r<3.11$ and it is in panel A ($k^*=4$) when $2.57<r<2.88$.

Second, when opting out is not generated by preferences based on single-round payoffs, other opting out rules are possible. For example, in the parallel universe where individuals prefer to be with Defectors, they might opt out when the number of Defectors is below the threshold $k^*$. For $m>2$, such an opting out rule no longer generates the identical game as the corresponding rule that applies this threshold to the number of Cooperators. However, there is a symmetry between these two games’ fitness functions and interior NE given by the following general result (proved in the electronic supplement) that holds for all $m\ge 2$.

#### **Theorem 1**

- **(a)** *Given* $N_C$, $N_D$ *and* $\{\tau _0,\tau _1,\dots , \tau _m\}$, *suppose that* $n_k$ *is the equilibrium group distribution. Then* $n'_k\equiv n_{m-k}$ *is the equilibrium group distribution given* $N'_C\equiv N_D$, $N'_D\equiv N_C$ *and* $\{\tau '_0\equiv \tau _m,\tau '_1\equiv \tau _{m-1},\dots , \tau '_m\equiv \tau _0 \}$. *Furthermore*, $\Pi '_C=(r+1)E-\Pi _D$ *and* $\Pi '_D=(r+1)E-\Pi _C$ *at these two distributional equilibria*.
- **(b)** *If* $N_C$, $N_D$ *is an interior NE for* $\{\tau _0,\tau _1,\dots , \tau _m\}$, *then* $N'_C\equiv N_D$, $N'_D\equiv N_C$ *is an interior NE for* $\{\tau '_0\equiv \tau _m,\tau '_1\equiv \tau _{m-1},\dots , \tau '_m\equiv \tau _0 \}$. *Furthermore, the stability at these two NE is opposite (i.e., if the interior original NE is stable, the primed one is unstable)*.

The symmetry contained in this Theorem is illustrated in Fig. 2 where interior NE in panels I, J for $\tau _0=\tau , \tau _1=...=\tau _4=1$ are the reflection (in $N_C=N/2$) of those in panels A, B for $\tau _0=...=\tau _3=1,\tau _4=\tau$. We see that these have interior NE for the same values of *r* (left panels) and for the same values of $\tau$ (right panels). Which opting out rule is better is debatable since opting out if there is a Defector in the group (panel A) has a higher level of cooperation at the stable interior NE but opting out if there is a Cooperator (panel I) has the larger domain of attraction. It should also be noted that there are opting out rules not shown in the figure (e.g., opt out if there are an odd number of Cooperators in your group) for which there are four interior NE (two stable and two unstable) for some $r<m=4$. The unstable NE and endpoints then partition the vertical axis into intervals. The domain of attraction of a stable NE is then the interval containing it in this partition.

Based on the above comparisons of opting out rules corresponding to a threshold level of Cooperators, it appears unlikely that PGG games generated by a single opting out rule can always outperform all others at promoting cooperation. By extensive numerical calculations, we examined PGG with opting out for groups of size $m=2,3,\dots ,10$ and maximum number of rounds played by a group fixed at $\tau =5$. For example, when $m=10$, there are $2^{11}=2048$ such PGG games opting-out. Table 1 summarizes, for each of these group sizes, the best opting out rule(s) in the sense that they have the minimum enhancement factor *r* at which a stable coexistence NE appears. For $m=2,3,4$, opting out if and only if some member of your group does not play your strategy outperforms all other opting out rules. As indicated in Table 1, this rule generates the PGG game where $\tau _0=\tau _m=5$ and $\tau _k=1$ for $k=1,2,\dots ,m-1$. These games correspond to Figs. 1G and 2K for $m=2$ and $m=4$ respectively where all groups disband immediately (i.e., after one round) unless all group members are cooperating, or all are defecting. For $m\ge 5$, there are two games, which are symmetric versions of each other, that provide the minimum enhancement factor (see Table 1). Again, homogeneous groups do not disband voluntarily. However, there is also a threshold level where heterogeneous groups disband immediately if the number of Cooperators is below this threshold (or above a threshold in the symmetric case). For example, when $m=5$, heterogeneous groups with fewer than 4 Cooperators disband immediately under the opting out rule 511155.

We also calculated numerically the best opting out rule when $\tau$ varies. For each group size $m=2,3,\dots ,6$, a minimum level exists for $\tau$ given in Table 2 for which the best rule is to opt out if and only if some member of your group does not play your strategy whenever $\tau$ is at or above this minimum. Since $\tau \ge 2$ in our repeated PGG games, this rule is best for all $\tau$ when $m=2,3$ as indicated in Table 2. On the other hand, when $m=4,5,6$, below the minimum given in Table 2, there are again two best opting out rules (that depend on $\tau$), which are symmetric versions of each other, analogous to Table 1 with 5 replaced by $\tau$ for $m\ge 5$. That is, if groups that do not disband voluntarily are expected to stay together for sufficiently many rounds, PGG games generated by opting out if and only if the group is heterogeneous outperform all others at promoting cooperation.

**Table 1:** The best opting-out rules for emergence of cooperation when the maximum number of rounds is five (i.e., $\tau =5$).

Group size *m*	Best opting-out rule(s)	Minimum enhancement factor *r*
2	515	1.2
3	5115	1.5
4	51115	2
5	511155	2.43
	551115	
6	5111555	2.88
	5551115	
7	51115555	3.34
	55551115	
8	511155555	3.80
	555551115	
9	5111555555	4.26
	5555551115	
10	51115555555	4.72
	55555551115

**Table 2:** The minimum number of rounds $\tau$ for which the best opting out rule has the form $\tau ,1,\dots ,1,\tau$, as a function of the group size *m*.

*m*	Minimum $\tau$
2	2
3	2
4	5
5	17
6	50

## Methods

### Fitness functions

To determine fitness functions in the classic single-round PGG with strategy set $\{C,D\}$, groups of size *m* are assumed to form at random from a large pool of individuals. The probability that a group has *k* Cooperators is then given by the binomial distribution with respect to the frequencies of C and D in the pool. From this distribution, the expected payoffs to C and D (i.e., their fitnesses) are determined as functions of these frequencies. Since the payoff to D is always greater than the payoff to C (since the enhancement factor is assumed to be smaller than the number of players in the group, i.e., $r<m$), the game solution is all D.

To analyze our *m*-player two-strategy game, these steps must be generalized to repeated games where the pool of individuals forming new groups depends on the number of rounds groups with *k* Cooperators are expected to play. When $m=2$, this has been done by Křivan and Cressman^12^ (see also^12,33–36^) through introducing and analyzing the pair formation dynamics. To extend their method to our *m*-player game, assume there is a population of Cooperators and Defectors of total size *N* where $N\gg m$ is very large (so that finite population effects can be ignored) and a multiple of *m*. There are then *N*/*m* groups. A group with *k* Cooperators plays an average $\tau _k$ rounds ($\tau _k\ge 1$) before disbanding following a Poisson process. That is, on average, $n_k/\tau _k$ of these groups disband after a given round where $n_k$ is the number of groups in the current round with *k* Cooperators. Thus, there are, on average, $n_C\equiv \frac{n_1}{\tau _1}+2 \frac{n_2}{\tau _2}+\dots + m \frac{n_m}{\tau _m}$ single Cooperators and $n_D\equiv m \frac{n_0}{\tau _0}+(m-1)\frac{n_1}{\tau _1}+(m-2) \frac{n_2}{\tau _2}+\dots + \frac{n_{m-1}}{\tau _{m-1}}$ single Defectors at the end of the current round. These singles immediately form new groups of *m* players at random.

Thus, the number of new groups with *k* Cooperators in the next round will be $p_k (\frac{n_0}{\tau _0}+\frac{n_{1}}{\tau _{1}}+\frac{n_{2}}{\tau _{2}}+ \dots +\frac{n_{m}}{\tau _{m}})$ where $p_k$ is the binomial distribution, i.e.,

3 $$\begin{aligned} p_k={{m}\atopwithdelims (){k}} \left( \frac{n_C}{n_C+n_D}\right) ^k \left( \frac{n_D}{n_C+n_D}\right) ^{m-k}. \end{aligned}$$

Furthermore, the number of groups with *k* Cooperators in the next round $n_k'$ is then given by the following discrete-time distribution dynamics (called the group formation dynamics)

4 $$\begin{aligned} n_{k}' =n_k -\frac{n_{k}}{\tau _{k}}+ p_k \left( \frac{n_{0}}{\tau _{0}}+\dots +\frac{n_{m}}{\tau _{m}} \right) ,\;\;\;\;k=0,\dots ,m. \end{aligned}$$

Our repeated PGG is then defined at fixed population size *N* by (pure) strategy space $\{C,D\}$ and an individual’s fitness given by his/her expected payoff per round at the equilibrium distribution of (4). Fitness is then a function of the total numbers $N_C$ and $N_D$ of Cooperators and Defectors, respectively, where $N_C+N_D=N$ is fixed. In fact, since the binomial distribution (3) depends only on the frequencies of single Cooperators and single Defectors, the equilibrium distribution is actually a function of the frequency of Cooperators and Defectors (i.e., it only depends on the population size *N* through a multiplicative factor). For example, let us consider a focal Cooperator. Since there are $k n_k$ Cooperators in groups with *k* Cooperators, the probability that a Cooperator is in a group of *k* Cooperators is $\frac{kn_k}{N_C}$ (where $N_C=\Sigma _{i=0}^m in_i$) and this Cooperator receives payoff $\pi _C(k)$. That is, the fitness $\Pi _C$ of a Cooperator defined as the average payoff is

5 $$\begin{aligned} \Pi _C=\frac{1}{N_C}\sum _{k=0}^{m}k n_k \pi _C(k)=\frac{1}{\sum _{i=0}^m i n_i}\sum _{k=0}^{m}k n_k \pi _C(k) \end{aligned}$$

where $n_k$ is at the equilibrium distribution of (4) for the total numbers $N_C$ and $N_D$ of Cooperators and Defectors. We note that $\Pi _C$ in (5) is indeterminate when $N_C=0$. In this case, we define $\Pi _C$ as the invasion fitness of a Cooperator in a population of all Defectors. This is given by $\pi _C(1)=\frac{rE}{m}$ and is also equal to $\lim _{N_C \rightarrow 0}\Pi _C$. The invasion fitness of D in an all C population is $\Pi _D=\pi _D(m-1)=(1+r)E-\frac{rE}{m}$. Similarly, the fitness $\Pi _D$ of a focal Defector is

6 $$\begin{aligned} \Pi _D=\frac{1}{N_D}\sum _{k=0}^{m}(m-k)n_k \pi _D(k)=\frac{1}{\sum _{i=0}^m (m- i) n_i}\sum _{k=0}^{m}(m-k)n_k \pi _D(k) \end{aligned}$$

since there are $(m-k) n_k$ Defectors in groups with *k* Cooperators.

### All groups play the same expected number of rounds

When all $\tau _k$ are equal to $\tau \ge 1$ as in the typical repeated PGG, then the unique equilibrium of (4) as a function of $N_C$ and $N_D$ is the binomial distribution scaled by the number of groups. That is,

$$\begin{aligned} n_k=\frac{N}{m}{{m}\atopwithdelims (){k}} \left( \frac{N_C}{N}\right) ^k \left( \frac{N_D}{N}\right) ^{m-k}. \end{aligned}$$

To see this, note that $\frac{n_{0}}{\tau _{0}}+\dots +\frac{n_{m}}{\tau _{m}}=\frac{N}{m\tau }$, the number of single Cooperators $n_C=\frac{N_C}{\tau }$ and the fraction of single Cooperators among all singles $\frac{n_C}{n_C+n_D}=\frac{N_C}{N}$. Then, at the equilibrium of (4), $n_k=p_k\frac{N}{m}$. Thus, since the equilibrium distribution is independent of $\tau$, the fitness functions are the same as those of the single round PGG (i.e., $\tau =1$) and so their stable NE are also the same.

### Analytic methods when $m=2$

For $m=2$ we can calculate the interior NE analytically. When $\tau _1^2-\tau _0 \tau _2= 0$ the distribution of pairs at the equilibrium of (4) is given by the Hardy–Weinberg proportions

7 $$\begin{aligned} n_0=\frac{(N-N_C)^2}{2 N},\;\; n_1=\frac{N_C(N-N_C)}{N},\;\;n_2=\frac{N_C^2}{2 N} \end{aligned}$$

and the difference $\Pi _C-\Pi _D$ reduces to $\frac{1}{2}E (r-2)$. This implies that the only NE is all D (respectively, all C) when $r<2$ (respectively, $r>2$), and the whole vertical segment $0\le N_C\le N$ is a set of NE when $r=2$.

When $\tau _0 \tau _2\ne \tau _1^2$, there exists a unique group distribution equilibrium

8 $$\begin{aligned} n_0=&\frac{\tau _1^2 (N-2 N_C)+2 \tau _0 \tau _2 \ (N_C-N)+\sqrt{\tau _1^4 (N-2 N_C)^2+4 N_C \tau _0 \tau _2 \tau _1^2 (N-N_C)}}{4 \left( \tau _1^2-\tau _0 \tau _2\right) },\\ n _1=&\frac{N \tau _1^2-\sqrt{\tau _1^4 (N-2 N_C)^2+4 N_C \tau _0 \tau _2 \tau _1^2 (N-N_C)}}{2 \left( \tau _1^2-\tau _0 \tau _2\right) },\\ n_2=&\frac{\tau _1^2 (2 N_C-N)-2 N_C \tau _0 \tau _2+\sqrt{\tau _1^4 (N-2 \ N_C)^2+4 N_C \tau _0 \tau _2 \tau _1^2 (N-N_C)}}{4 \left( \tau _1^2-\tau _0 \tau _2\right) }. \end{aligned}$$

When $\tau _0 \tau _2>\tau _1^2$, it is straightforward to show that $n_0$ and $n_2$ in (8) are larger than the Hardy-Weinberg proportions in (7). That is, there is positive assortment of Cooperators and of Defectors. Similarly, there is negative assortment when $\tau _0 \tau _2<\tau _1^2$. Interior NE exist and are given by

9 $$\begin{aligned} N_C=\frac{1}{2} N \left( 1\pm \sqrt{ \frac{(r-1)^2 \tau _0 \tau _2-\tau _1^2}{(r-1)^2 (\tau _0 \tau _2-\tau _1^2)}}\right) \end{aligned}$$

when $\tau _0 \tau _2>\tau _1^2$ (respectively, $\tau _0 \tau _2<\tau _1^2$) if and only if

10 $$\begin{aligned} 1+ \frac{\tau _1}{\sqrt{\tau _0 \tau _2}}\le r<2 \;\;\left( \text {respectively,} \;\;2<r\le 1+ \frac{\tau _1}{\sqrt{\tau _0 \tau _2}}\right) . \end{aligned}$$

From (8) and (9), we observe that the equilibrium group distribution and the NE depend on $\tau _0$ and $\tau _2$ only through their product $\tau _0\tau _2$. In particular, since $\tau _0\tau _2=\tau$ and $\tau _1=1$ for opting out if your opponent Defects (i.e., the group disbands if at least one member Defects) as well as for opting out if your opponent Cooperates, these two PGG are identical games (i.e., they have the same fitness functions and NE). Furthermore, for these two games, the interior NE emerges when $1+ \frac{1}{\sqrt{\tau }}< r$ (i.e., when $\tau > (\frac{1}{r-1})^2$) if $r<2$.

From (10), we also observe that the minimum (respectively, maximum) value of *r* for which the interior NE exists is achieved when $\tau _1=1$ (respectively, $\tau _1=\tau$) and $\tau _{0}=\tau _{2}=\tau$ (respectively, $\tau _{0}=\tau _{2}=1$). Criterion (1) and Criterion (2) follow from this minimum value. From (9), the domain of attraction of the stable interior NE is the open interval $\frac{1}{2}N\left( 1-\sqrt{ \frac{(r-1)^2 \tau _0 \tau _2-\tau _1^2}{(r-1)^2 (\tau _0 \tau _2-\tau _1^2)}}\right)< N_C<N$. Criterion (3) follows from the strict inequality $\sqrt{ \frac{(r-1)^2 \tau _0 \tau _2-\tau _1^2}{(r-1)^2 (\tau _0 \tau _2-\tau _1^2)}}<\sqrt{ \frac{(r-1)^2 \tau ^2-1}{(r-1)^2 (\tau ^2-1)}}$ which holds unless $\tau _0=\tau _2=\tau$ and $\tau _1=1$. Finally, since $\frac{1}{2} N \left( 1+\sqrt{ \frac{(r-1)^2 \tau _0 \tau _2-\tau _1^2}{(r-1)^2 (\tau _0 \tau _2-\tau _1^2)}}\right) < \frac{1}{2} N \left( 1+\sqrt{ \frac{(r-1)^2 \tau ^2-1}{(r-1)^2 (\tau ^2-1)}}\right)$ unless $\tau _0=\tau _2=\tau$ and $\tau _1=1$, opting out if your group is not homogeneous has the higher level of cooperation at the stable interior NE (Criterion (4)).

Note that, when $r=2$, there are no interior NE if $\tau _0 \tau _2\ne \tau _1^2$ (Fig. 1, left panels). To see this, we observe that

$$\begin{aligned} \Pi _C-\Pi _D=&\frac{E \left( 4 (N-N_C)N_C \left( \tau _1^2-\tau _0 \tau _2\right) -2 N \left( N \tau _1^2-\sqrt{\tau _1^4 (N-2 N_C)^2+4 N_C \tau _0 \tau _2 \tau _1^2 (N-N_C)}\right) \right) }{4 \ (N-N_C)\left( \tau _1^2-\tau _0 \tau _2\right) }. \end{aligned}$$

Then $\Pi _C \ne \Pi _D$ if $N_C$ and $N_D=N-N_C$ satisfy $0<N_C,N_D<N$ since

$$\begin{aligned} 4(N-N_C)N_C(\tau _1^2-\tau _0 \tau _2)>2N \left( N \tau _1^2-\sqrt{\tau _1^4 (N-2 N_C)^2+4 N_C \tau _0 \tau _2 \tau _1^2 (N-N_C)}\right) \end{aligned}$$

which is equivalent with $4 N_C^2 \left( \tau _1^2-\tau _0 \tau _2\right) ^2 \ (N_C-N)^2/N^2>0.$ On the other hand, all D is a NE since then $\Pi _D=\pi _D(0)=E$ and the invasion fitness of C is $\Pi _C=\pi _C(1)=\frac{rE}{m}=E$. All C is also a NE since $\Pi _D=\pi _D(m-1)=(1+r)E-\frac{rE}{m}=2E$ and $\Pi _C=\pi _C(m)=2E$. Neither of these are strict NE since $\Pi _C = \Pi _D$ at all D and at all C. This same argument shows that all D (respectively, all C) is a strict NE if and only if $r<m$ (respectively, $r>m$) when $m=2$.

### Numeric methods when $m>2$

When $m>2$, neither the equilibrium group distribution of (4) nor coexistence NE can be calculated analytically. Since these NE satisfy $\Pi _C=\Pi _D$ where $\Pi _C$ and $\Pi _D$ are given by (5) and (6), respectively, together with equations describing the equilibrium group distribution $\frac{n_{k}}{\tau _{k}}=p_k (\frac{n_{0}}{\tau _{0}}+\dots +\frac{n_{m}}{\tau _{m}}),\;\;\;\;k=0,\dots ,m$ (see subsection Fitness functions in “Methods” section), we solve these equations numerically in Mathematica^37^ assuming total population size $N=100$. In particular, plots in Fig. 2 for $m=4$ were obtained by solving these equations for unknowns *r*, $n_0$, $n_1$, $n_2$, $n_3$, $n_4$ for each $N_C$ between 0 and 100, with step 1 (i.e., $N_C=0,1,2,3,4,5,6,....,100$). Figures 1 and 2 show that there can be several NE for a given enhancement factor *r*. However, only those corresponding to the solid line are stable with respect to evolutionary dynamics. For fixed *r* and $\tau$, these NE satisfy the condition that $\Pi _C>\Pi _D$ for $N_C$ slightly below the NE and $\Pi _C<\Pi _D$ for $N_C$ slightly above since nearby trajectories of the evolutionary dynamics then converge to such a NE. Based on these stability conditions, stable NE can be determined analytically for $m=2$. When $m>2$, the fitness differences $\Pi _C-\Pi _D$ are examined numerically instead.

To generate Table 1, we also calculated the minimum enhancement factor *r* at which the interior NE occurs for all PGG with opting out and at most five rounds in a game for each group size $m=3,\dots ,10$. For example, when $m=4$, the minimum *r* was calculated numerically over $N_C=0,\dots ,100$ for all combinations of $\tau _0,\dots ,\tau _4$ where $\tau _i\in \{1,5\}$, $i=0,\dots ,4$.

## Discussion

We conclude that, to promote cooperation in the PGG when the expected time a group remains together depends on the number of Cooperators in the group, it is best if players base their decision to opt out on the homogeneity of their current group in order that the group disbands after one round if and only if it is heterogeneous. In particular, players then resist the appeal of basing their decision to opt out on single-round payoffs since this approach suggests they should be more prone to opt out as the number of Defectors in their group increases. Although there is evidence that players in game experiments on social dilemmas^9,15^ do opt out more against Defectors, there are also instances where intolerance to individuals who are not like-minded is an important aspect of individual behavior. For example, Aksoy^38^ found that the level of cooperation with a like-minded individual (called an in-group member) was higher than with an out-group member. Similarly, Koger et al.^29^ analyzed cooperation between party factions in the United States, finding that there was a considerable amount of cooperation among different factions of the Democratic Party and, to a lesser extent, among different factions of the Republican Party.

To continue the left-right analogy started at the end of Section Opting out in the two-player public goods game, suppose that, for the sake of argument, the two types of behaviors are called left and right and that they correspond to Cooperate and Defect respectively in an *m*-player PGG. That is, an individual on the right does better than one on the left when the right-left distribution of other group members is fixed while the group does best if it consists of all left individuals. Then, if the expected time that a group stays together does not depend on its distribution of right-left individuals, the right wins (i.e., the stable NE is that all individuals become right) and everyone does poorly. On the other hand, individuals on the right often prefer like-minded individuals. For example, Altemeyer^39^ shows that Republicans exhibit the traits of right-wing authoritarians much more that Democrats and, according to Wikipedia^40^, such authoritarians are “hostile and punitive” towards people who, unlike them, do not adhere to societal norms. Our results show that such a preference may lead to a stable mixture of left and right individuals in the population. In fact, suppose this preference is so strong that right-minded individuals will force the group to disband if it contains any left members. This intolerance of the left overrides payoff consequences for these individuals in that their payoff would be higher if they were part of a group that contains some left members. Then the left can actually win, especially if right groups tend to stay together for a long time $\tau$, in that the population evolves to most individuals becoming left (Figs. 1F,H, 2J,L). This conclusion does not depend on whether the left opts out (specifically, it is independent of the expected number of rounds an all left group stays together). That is, intolerance of the right leads to its downfall. This Achilles heel of the right is even more pronounced if individuals on the left also prefer to be with like-minded individuals (compare Fig. 1 panel F to H and Fig. 2 panel J to L) since the left then wins for smaller values of $\tau$.

Interestingly, the left also wins if they are intolerant (i.e., left individuals force their group to disband if it contains any right individuals) independent of whether right individuals opt out. In fact, for two-player PGG, the same outcome occurs when it is only all left groups that stay together (Fig. 1A,B) as when it is only all right groups (Fig. 1E,F). On the other hand, for four-player PGG, the invasion of the all D population configuration by the left occurs more readily when only the all right group stays together (Fig. 2J) compared to when only the all left group stays together (Fig. 2B).

Our analysis of the effects of opting out in the repeated PGG has concentrated on examining the NE structure of two-strategy games where each player is either a Cooperator (i.e., plays C for the entire game) or a Defector (D) and all Cooperators have the same opting out rule as do all Defectors. We are particularly interested in how these rules affect the level of cooperation at the stable NE of these games. For two-player PGG (i.e., $m=2$), our analytic results show that opting out against Defect leads to stable coexistence of C and D in the population. This outcome is similar to previous theoretical and empirical studies for the repeated Prisoner’s dilemma (PD) game (e.g.,^9–12,15^) and consistent with the finding that group partners are more cooperative when committed to each other in two-player repeated PGG^16^. On the other hand, it is also shown that opting out if your partner does not share your strategy leads to the highest possible level of cooperation due to the increased positive assortment of CC and DD pairs.

Our extensions of these results to multi-player (i.e., $m>2$) PGG, where analytic methods are no longer possible, are of equal or greater importance. As shown by extensive numerical calculations, when groups that do not disband immediately are expected to stay together for a large number of rounds, the repeated PGG game with opting out that best promotes cooperation now emerges when all heterogeneous *m*-player groups immediately disband, an outcome that occurs if Defectors are completely intolerant of Cooperators. As far as we are aware, this theoretical prediction has not been tested either in game or other social experiments and we hope this article will motivate researchers to do so.

## Supplementary information

Supplementary Information.
