# Supplementary material for: Defectors’ intolerance of others promotes cooperation in the repeated public goods game with opting out
Source: Sci Rep. 2020 Nov 11;10:19511. doi: 10.1038/s41598-020-76506-3 (PMC7659018; doi:10.1038/s41598-020-76506-3)
Supplement: Supplementary file 1 — Supplementary Information. [file 41598_2020_76506_MOESM1_ESM.pdf]

Defectors' intolerance of others promotes cooperation in the repeated public goods game with opting out

Vlastimil Křivan[1,3,*] **and Ross Cressman**[2,+]

[1]Faculty of Science, University of South Bohemia, Department of Mathematics, České Budějovice, 370 05, Czech Republic
[2]Wilfrid Laurier University, Department of Mathematics, Waterloo, Ontario, Canada
[3]Czech Academy of Sciences, Biology Centre, České Budějovice, 370 05, Czech Republic
[*]vlastimil.krivan@gmail.com
[+]rcressman@wlu.ca

S1. Proof of Theorem 1.

First we prove part (a) of Theorem 1. Under the conditions of part (a), $n'_C = \frac{m'_1}{\tau'_1} + \cdots + m\frac{n'_m}{\tau'_m} = \frac{n_{m-1}}{\tau_{m-1}} + \cdots + m\frac{n_0}{\tau_0} = n_D$ and $n'_D = n_C$. Also

$$p'_k = \binom{m}{k}\left(\frac{n'_C}{n'_C+n'_D}\right)^k\left(\frac{n'_D}{n'_C+n'_D}\right)^{m-k} = \binom{m}{m-k}\left(\frac{n_D}{n_C+n_D}\right)^k\left(\frac{n_C}{n_C+n_D}\right)^{m-k} = p_{m-k}$$

and $p'_{m-k} = p_m$. Then $\frac{n'_k}{\tau'_k} = p'_k\left(\frac{n'_0}{\tau'_0} + \ldots + \frac{n'_m}{\tau'_m}\right)$ i.e., $\frac{n_{m-k}}{\tau_{m-k}} = p_{m-k}(\frac{n_m}{\tau_m} + \ldots + \frac{n_0}{\tau_0})$. Furthermore, $N'_C = n'_1 + 2n'_2 + \cdots + mn'_m = n_{m-1} + 2n_{m-2} + \cdots + mn_0 = N_D$ and $N'_D = N_C$. Thus, $n'_0, \cdots, n'_m$ is the group equilibrium distribution for $N'_C = N_D$ and $N'_D = N_C$.

Now

$$\Pi_C = \frac{1}{N_C}\sum_{k=0}^{m} kn_k\pi_C(k) = \frac{1}{N'_D}\sum_{k=0}^{m} kn'_{m-k}\pi_C(k) = \frac{1}{N'_D}\sum_{k=0}^{m}(m-k)n'_k\pi_C(m-k),$$

$$\pi_C(m-k) = (m-k)\frac{rE}{m} = rE - \frac{krE}{m},$$

$$\pi_D(k) = E + \frac{krE}{m},$$

$$\pi_C(m-k) = (r+1)E - \pi_D(k).$$

Thus

$$\Pi_C = \frac{1}{N'_D}\sum_{k=0}^{m}(m-k)n'_k\big((r+1)E - \pi_D(k)\big) = \frac{1}{N'_D}\left(\sum_{k=0}^{m}(m-k)n'_k\right)(r+1)E - \Pi'_D = (r+1)E - \Pi'_D$$

since $\Sigma_k(m-k)n'_k = N'_D$. Similarly, $\Pi_D = (r+1)E - \Pi'_C$.

Second, we prove part (b) of Theorem 1. The equilibrium group distribution at (N_C, N_D) corresponds to an interior NE of the PGG based on $\tau_0, \cdots, \tau_m$ if and only if $0 < N_C < N$ and $\Pi_C = \Pi_D$. Since $\Pi'_C - \Pi'_D = (r+1)E - \Pi_D - ((r+1)E - \Pi_C) = \Pi_C - \Pi_D$ at the two distributional equilibria of part (a), $\Pi_C = \Pi_D$ if and only if $\Pi'_C = \Pi'_D$. That is (N_C, N_D) is an interior NE if and only if (N'_C, N'_D) is as well.

Furthermore, an interior NE at (N^*_C, N^*_D) is stable [1, 2, 3, 4] if and only if $(\Pi_C - \Pi_D)(N_C - N_D) < 0$ for all N_C sufficiently close (but not equal) to N^*_C. At the two distributional equilibria of part (a), $(\Pi'_C - \Pi'_D)(N'_C - N'_D) = (\Pi_C - \Pi_D)(N_D - N_C)$ and so $(\Pi'_C - \Pi'_D)(N'_C - N'_D) < 0$ if and only if $(\Pi_C - \Pi_D)(N_C - N_D) > 0$. Thus, (N^*_C, N^*_D) is stable if and only if $(N^{*\prime}_C, N^{*\prime}_D)$ is unstable.

References

1. M. Broom and J. Rychtář. *Game-Theoretical Models in Biology*. CRC Press, Taylor & Francis Group, Boca Raton, FL, 2013.

2. R. Cressman. *Evolutionary dynamics and extensive form games*. The MIT Press, Cambridge, MA, 2003.
3. J. Hofbauer and K. Sigmund. *Evolutionary games and population dynamics*. Cambridge University Press, Cambridge, UK, 1998.
4. J. Maynard Smith and G. R. Price. The logic of animal conflict. *Nature*, 246:15–18, 1973.
